# Epidemiology of resistant gram-negative bacteria in nursing homes

**DOI:** 10.1017/ice.2022.225

**Published:** 2023-09

**Authors:** John P. Mills, Julia Mantey, Marco Cassone, Keith S. Kaye, Lona Mody

**Affiliations:** 1 Division of Infectious Diseases, Department of Medicine, Rutgers Robert Wood Johnson Medical School, New Brunswick, New Jersey; 2 Division of Geriatric and Palliative Medicine, Department of Internal Medicine, University of Michigan, Ann Arbor, Michigan; 3 Veterans’ Affairs Ann Arbor Healthcare System, Ann Arbor, Michigan

## Abstract

**Background:**

Resistant gram-negative bacteria (R-GNB) colonization in nursing home patients can cause clinical infection and intrafacility transmission. Limited data exist on the roles of age and function on R-GNB colonization.

**Methods:**

A secondary data analysis was performed from a cohort study of 896 patients admitted to 6 Michigan nursing homes between November 2013 and May 2018. Swabs obtained upon enrollment, weekly for 1 month, then monthly until nursing home discharge from 5 anatomical sites were cultured for GNB. R-GNB were defined as resistant to ciprofloxacin, ceftazidime, or imipenem. Patients with growth of the same R-GNB as the initial positive visit, from any anatomical site at any subsequent visit, were considered persistently colonized. Demographic data, antibiotic use, device use, and physical self-maintenance scales (PSMSs) were obtained upon enrollment. Characteristics were compared between patients with R-GNB colonization versus those without, and those with persistent R-GNB colonization versus those with spontaneous decolonization.

**Results:**

Of 169 patients with a positive R-GNB culture and ≥2 subsequent study visits, 89 (53%) were transiently colonized and 80 (47%) were persistently colonized. Compared to uncolonized patients, persistent and transient R-GNB colonization were associated with higher PSMS score: 1.14 (95% confidence interval or CI, 1.05–1.23; *P* = .002) and 1.10 (95% CI, 1.01–1.19; *P* = .023), respectively. Persistent colonization was independently associated with longer duration of nursing home stay (1.02; 95% CI, 1.01–1.02; *P* < .001). Higher readmission rate among persistently colonized patients was observed on unadjusted analysis.

**Conclusions:**

Persistent R-GNB colonization is associated with younger age, functional disability, and prolonged length of nursing home stay. In-depth longitudinal studies to understand new acquisition and transmission dynamics of R-GNB in nursing homes are needed.

Infections due to antibiotic-resistant organisms (AROs) disproportionally affect older adults, particularly nursing home patients, who have higher rates of chronic illness, debility, and healthcare system exposure.^1^ Drug-resistant gram-negative bacteria (R-GNB) are particularly problematic due to limited available treatment options as well as their propensity for prolonged gastrointestinal colonization and potential for widespread dissemination.^2,3^

A significant proportion of urinary tract infections in nursing homes are caused by R-GNB.^4^ The dynamics of R-GNB intestinal colonization are unclear, with some studies suggesting stable persistence and others suggesting frequent occurrences of acquisition and spontaneous decolonization as well as the highly variable duration of intestinal carriage.^5,6^ Although colonization with R-GNB precedes infection, it is currently unclear which factors influence colonization status, the frequency with which colonized patients develop R-GNB infections, and the consequences of colonization and infection status on subsequent health outcomes such as functional status and hospital admission among nursing home patients. We sought to determine clinical factors associated with persistent versus transient R-GNB colonization to identify potential targets for future intervention.

## Methods

### Study population and design

Secondary data analysis was performed utilizing data from a prospective cohort study examining longitudinal ARO colonization status of 896 patients admitted to 6 Michigan nursing homes between November 2013 and July 2018. Patients were enrolled within 14 days of nursing home admission and were visited thereafter on days 7, 14, 21, 30, and then monthly for up to 6 months. Patients receiving hospice care were excluded. Informed consent was obtained from patients or his or her legal proxy. The study was approved by the University of Michigan Institutional Review Board.

Clinical data were obtained by trained research staff. Demographic data (age, sex, race), comorbidities (including Charlson comorbidity index), and cognitive status were obtained upon enrollment. Antibiotic use in the prior 30 days, hospitalization, presence of indwelling devices, functional status, and presence of clinical infections were obtained at each study visit. Functional status was collected utilizing the Physical Self-Maintenance Scale (PSMS). Antibiotics were further dichotomized as high- versus low-risk agents for acquisition of ARO or *Clostridioides difficile* infection (CDI) based on previously validated metrics.^7^ High-risk agents consisted of fluoroquinolones, third–fifth generation cephalosporins, β-lactam/β-lactamase combinations, carbapenems, and lincosamines. Clinical infections (urinary tract infection [UTI], pneumonia, skin soft-tissue infection [SSTI], and CDI) were defined based on diagnosis by the treating physician of record at the nursing home.

### Specimen collection and microbiology

At each study visit, sterile swabs (BactiSwab, Remel, Lenexa, KS) were used to obtain samples from 5 anatomical sites (ie, perirectal area, groin, nares, oropharynx, and dominant hand), as well as any wounds, indwelling urinary catheters, or feeding tubes. All patients provided consent. Swabs from the hand were enriched overnight in brain–heart infusion broth at 36°C to improve culture sensitivity, considering lower bacterial burden from hands compared to anatomical sites. All swabs were plated on MacConkey agar. Genus and species of colonies were identified using bioMerieux analytical profile index (API, bioMerieux, Marcy l’Etoile, France). Antimicrobial susceptibility testing by disk diffusion was performed according to Clinical and Laboratory Standards Institute (CLSI) guidelines (M100-S23). Gram-negative bacilli were considered antimicrobial resistant (R-GNB) if nonsusceptible to ciprofloxacin, ceftazidime, or imipenem for all species except *Proteus mirabilis*. *P. mirabilis* isolates were considered R-GNB if they were nonsusceptible to ciprofloxacin, ceftazidime, or meropenem.

### Statistical analysis

Patients were considered colonized on a study visit if an R-GNB was detected from any swab collected. Patients were not excluded from analysis based on swabs collected. For the analysis of transient versus persistent R-GNB colonization, patients with <2 subsequent visits after initial R-GNB isolation were excluded. Among patients with ≥2 visits following initial colonization, those with all sampled body-site cultures negative for repeated R-GNB growth in their final 2 visits were considered transiently colonized. Those with repeated growth of the same R-GNB over multiple visits, from any anatomical site, were considered persistently colonized. For patients cocolonized with multiple R-GNB, the most frequently isolated species was selected for analysis.

Characteristics were compared between patients who were never colonized with R-GNB, those with transient R-GNB colonization, and those with persistent R-GNB colonization. We used the Pearson χ^2^ test and the Fisher exact test (where tables produced expected cell counts <10) to compare categorically defined characteristics among the 3 groups. We used the Wilcoxon Kruskal-Wallis test for continuous variables and multinomial regression to assess unadjusted associations between predictors of interest and persistence of colonization. Variables with *P* < .10, and those with biologic plausibility were considered for inclusion in the final model. Multinomial logistic regression using backward stepwise selection was utilized to identify factors associated with transient and persistent R-GNB colonization. Models were adjusted for confounding and were then assessed for collinearity and clustered by nursing home to adjust for facility-level differences. All statistical analysis was performed using Stata version 15.0 software (StataCorp, College Station, TX).

## Results

### R-GNB colonization status of total cohort

**Fig. 1.**
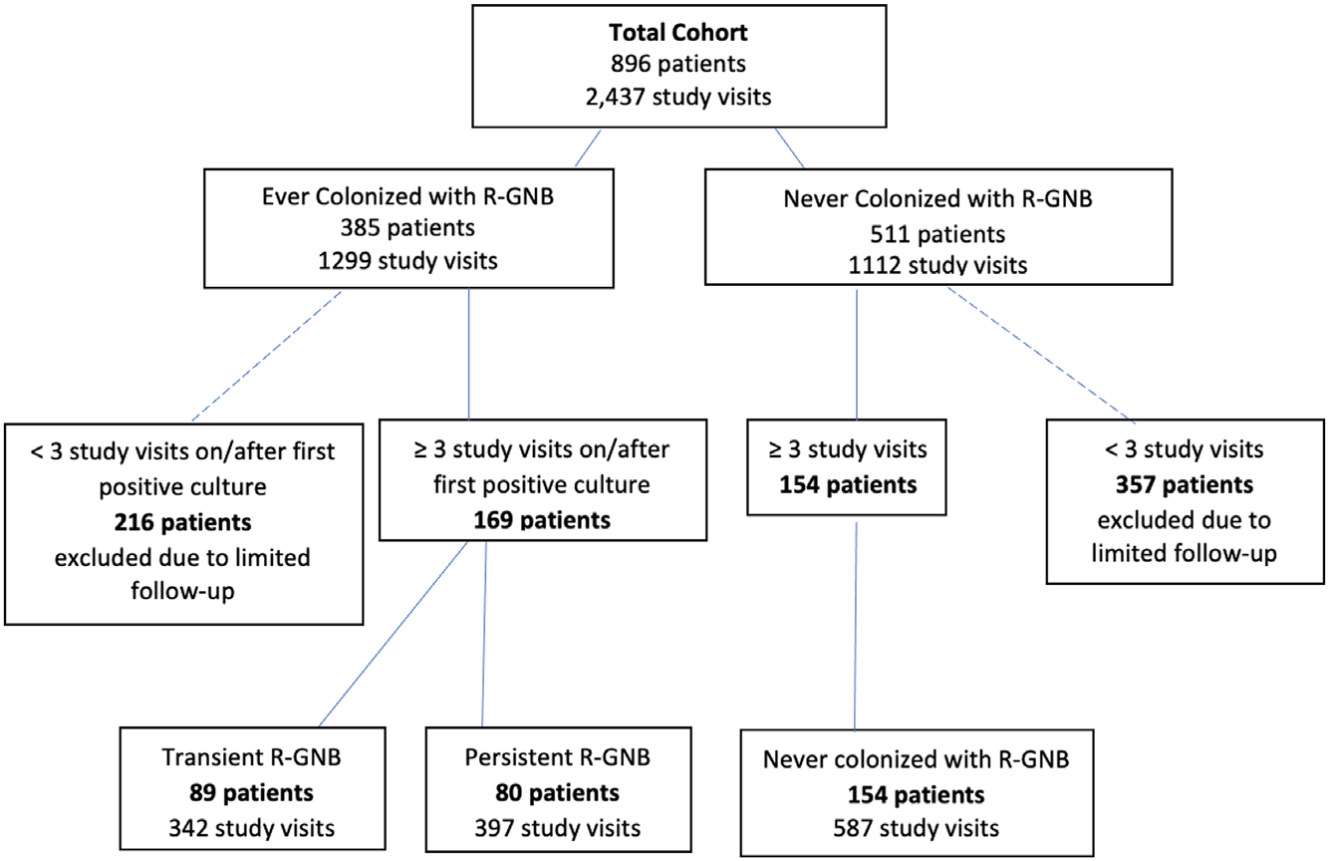
Patient cohorts by R-GNB colonization status.

The cohort consisted of 896 patients comprising 2,437 total study visits. R-GNB were isolated from ≥1 body site on at least 1 visit in 385 (43%) of 896 patients. Of 169 patients with a positive R-GNB culture and ≥ 2 subsequent study visits, 89 patients (53%) were considered to have transient R-GNB colonization and 80 patients (47%) were considered to have persistent R-GNB colonization (Fig. 1).

### Microbiology and anatomical sites of R-GNB colonization of study cohort

The most common body sites with R-GNB isolated were perirectal (n = 86, 66%) and groin (n = 73, 43%) (Supplementary Table 1 online). Colonization at ≥2 body sites at initial detection occurred in 39% of persistently colonized patients and 21% of transiently colonized patients. At the patient level, the 3 most identified organisms were *Escherichia coli* (the most frequently detected organism in 57 patients, 33.7%); *Proteus mirabilis* (the dominant organism in 19 patients, 11.2%); and *Klebsiella pneumoniae* (the dominant organism in 18 patients, 10.7%). *E. coli* and *P. mirabilis* were predominantly resistant to ciprofloxacin (22 of 25 patients or 88% and 17 of 17 patients or 100%) on first detection in patients who were persistently colonized. On first detection, *Klebsiella pneumoniae* was predominantly resistant to ceftazidime (9 of 13, 69%) and ciprofloxacin (8 of 13, 62%) (Supplementary Table 2 online). Persistently colonized patients had the same organism detected for a median of 42 days (IQR, 15–90) (Supplementary Table 3 online).

### Characteristics of uncolonized, transiently colonized, and persistently colonized patients

Sex, median age, and Charlson comorbidity index scores were similar between the uncolonized, transiently colonized, and persistently colonized groups (Table 1). Recent antibiotic use was also similar; >50% of patients in all 3 groups had exposure within the prior 30 days. Persistently colonized patients had significantly longer lengths of stay at a nursing home (*P* < .001) and higher rates of rehospitalization compared to uncolonized and transiently colonized patients (*P* = .001).

**Table 1.** Patient Demographics by R-GNB Colonization Status

Variable	Total Cohort (n=323)	Never R-GNB Colonization (n=154)	Transient R-GNB Colonization (n=89)	Persistent R-GNB Colonization (n=80)	*P* Value
Age, median y (IQR)	75 (65–85)	77 (66–86)	74 (64–85)	75.5 (65–83.5)	.464^a^
Sex, male, no. (%)	130 (40.3)	67 (43.5)	32 (36.0)	31 (38.8)	.488^b^
Race, nonwhite, no. (%)	146 (45.2)	62 (40.3)	45 (50.6)	39 (48.8)	.228^b^
Charlson comorbidity index, median score (IQR)	2 (1–4)	2 (1–4)	2 (1–4)	2 (1–4)	.905^a^
Dementia no./total (%)^c^	70/322 (21.7)	25/153 (16.3)	22/89 (24.7)	23/80 (28.8)	.067^b^
**PSMS score, median (IQR)** ^**c**^	14 (12–18) (N=321)	13 (11–17) (N=152)	16 (12–19) (N=89)	17 (14–21) (N=80)	**<.001** ^**a**^
PSMS toilet	2 (1–4)	2 (1–3)	3 (1–5)	3 (2–5)	**<.001** ^**a**^
PSMS feeding	1 (1–2)	1 (1–1)	1 (1–2)	1 (1–2)	**.004** ^**a**^
PSMS dressing	3 (2–4)	3 (2–3)	3 (2–4)	3 (3–4)	**<.001** ^**a**^
PSMS grooming	2 (1–3)	2 (1–2)	2 (1–3)	2 (2–3)	**<.001** ^**a**^
PSMS ambulation	3 (3–4)	3 (3–3)	3 (3–3)	3.5 (3–4)	**<.001** ^**a**^
PSMS bathing	3 (2–3)	3 (2–3)	3 (2–3)	3 (2.5–4)	**<.001** ^**a**^
Indwelling device upon admission, no. (%)	58 (18.0)	17 (11.0)	18 (20.2)	23 (28.8)	**.003** ^**b**^
Urinary catheter, no. (%)	43 (13.3)	14 (9.1)	15 (16.9)	14 (17.5)	.102^b^
Feeding tube, no. (%)	17 (5.3)	3 (2.0)	4 (4.5)	10 (12.5)	**.004** ^d^
Open wounds, no. (%)^e^	63/322 (19.6)	22/153 (14.4)	19/89 (21.4)	22/80 (27.5)	.050^b^
Hospital LOS prior to NH admission, median (IQR)	6 (3–10) (N=308)	6 (4–10) (N=147)	5 (3–11) (N=87)	6 (3–9) (N=74)	.359^a^
Duration of study follow-up, median (IQR)	28.5 (27–62)	28 (22–33)	30 (27–81)	60.5 (|28–175.5)	**<.001** ^**a**^
Duration prior to first colonization/study exit, median (IQR)	14 (0–28)	28 (22–32)	0 (0–9)	0 (0–6)	**<.001** ^**a**^
Antibiotics upon enrollment (any), no./total (%)	178/308 (57.8)	83/149 (55.7)	48/84 (57.1)	47/75 (62.7)	.603^b^
High-risk antibiotics, no./total (%)^f^	140/309 (45.3)	67/149 (45.0)	39/84 (46.4)	34/76 (44.7)	.971^b^
**Outcomes, no. (%)**					
Rehospitalization	86 (26.6)	28 (18.2)	26 (29.2)	32 (40.0)	**.001** ^**b**^
Clinical Infection (after first colonization), any	101 (31.3)	38 (24.7)	28 (31.5)	35 (43.8)	.074^b^

Note. R-GNB, resistant gram-negative bacteria; IQR, interquartile range; PSMS, physical self-maintenance scales; LOS, length of stay; NH, nursing home. Bold indicates statistical significance.

a Significance evaluated using Kruskal-Wallis test.

b Significance evaluated using Pearson’s χ^2^ test.

c N=316 for PSMS measures.

d Significance evaluated using the Fisher exact test.

e N=317 for open wounds.

f N=304 for antibiotics data.

### Outcomes among uncolonized, transiently colonized, and persistently colonized patients

On bivariate analysis, both transiently and persistently colonized patients had higher rates of rehospitalization when compared to uncolonized patients (Table 1). Overall rates of clinical infections were similar between the 3 groups. However, persistently colonized patients had higher rates of UTI diagnosis (25.0%) compared to transiently colonized patients (14.6%) and uncolonized patients (13.6%).

### Predictors of persistent R-GNB colonization

In unadjusted multinomial regression models, persistent and transient colonization showed similar patterns of association with predictors of interest, including higher PSMS (indicating lower functional status) and presence of indwelling devices (Table 2). Persistent R-GNB colonization was additionally associated with open wounds (odds ratio [OR], 2.26; 95% confidence interval [CI], 1.07–4.77; *P* = .033) compared with noncolonization. Lack of independence with grooming and ambulation was associated with persistent R-GNB colonization compared to transient colonization. Total antibiotic use and exposure to high-risk antibiotic classes were frequent in all 3 groups and were not associated with transient or persistent R-GNB colonization.

**Table 2.** Unadjusted Multinomial Analysis of Patient Characteristics Associated with Colonization Status

Variable	Transient vs Uncolonized RRR (95% CI)	*P* Value	Persistent vs Uncolonized RRR (95% CI)	*P* Value	Persist vs Transient RRR (95% CI)	*P* Value
Age	0.99 (0.98–1.01)	.168	0.99 (0.96–1.01)	.292	1.00 (0.98–1.02)	.737
Sex, male	0.73 (0.47–1.14)	.166	0.82 (0.51–1.31)	.412	1.13 (0.78–1.64)	.531
Race, nonwhite	1.52 (0.91–2.54)	.111	1.41 (0.60–3.31)	.428	0.93 (0.62–1.39)	.724
Charlson comorbidity index	1.02 (0.98–1.06)	.253	1.02 (0.94–1.20)	.654	0.99 (0.90–1.10)	.914
Dementia	1.68 (0.67–4.19)	.265	2.07 (0.88–4.87)	.097	1.23 (0.54–2.81)	.625
**PSMS**						
PSMS score	**1.10 (1.05–1.16)**	**<.001**	**1.17 (1.20–1.24)**	**<.001**	1.06 (0.99–1.13)	.081
PSMS, amb	1.41 (0.97–2.04)	.068	**3.48 (1.48–8.17)**	**.004**	**2.46 (1.25–4.87)**	**.009**
PSMS, groom	**1.48 (1.17–1.89)**	**.001**	**1.77 (1.31–2.37)**	**<.001**	1.19 (0.95–1.50)	.137
PSMS, dress	**1.57 (1.21–2.02)**	**.001**	**1.87 (1.37–2.54)**	**<.001**	1.19 (0.84–1.68)	.322
PSMS, toilet	**1.31 (1.12–1.53)**	**.001**	**1.39 (1.22–1.58)**	**<.001**	1.06 (0.89–1.26)	.494
PSMS, bath	**1.67 (1.20–2.33)**	**.003**	**2.17 (1.57–3.00)**	**<.001**	1.30 (0.75–2.26)	.347
PSMS, feed	1.21 (0.90–1.63)	.202	**1.60 (1.18–2.16)**	**.002**	**1.32 (1.02–1.71)**	**.038**
Indwelling device on admission	**2.04 (1.15–3.62)**	**.014**	**3.25 (1.86–5.68)**	**<.001**	**1.59 (1.07–2.38)**	**.023**
Urinary catheter	**2.03 (1.05–3.91)**	**.035**	**2.12 (1.09–4.13)**	**.027**	1.05 (0.57–1.94)	.885
Feeding tube	**2.37 (1.13–4.95)**	**.022**	**7.19 (1.90–27.22)**	**.004**	3.04 (0.73–12.58)	.126
Open wounds	1.62 (0.75–3.50)	.223	**2.26 (1.07–4.77)**	**.033**	1.40 (0.60–3.24)	.435
Hospital LOS prior to NH	1.00 (0.96–1.05)	.827	1.01 (0.99–1.04)	.380	1.01 (0.98–1.04)	.677
Duration of study follow-up	**1.01 (1.01–1.02)**	**<.001**	**1.02 (1.01–1.03)**	**<.001**	**1.01 (1.00–1.01)**	**<.001**
Days to first positive symptom or diagnosis	**0.92 (0.87–0.98)**	**.007**	**0.90 (0.86–0.95)**	**<.001**	0.98 (0.92–1.04)	.574
Antibiotics upon enrollment (any)	1.06 (0.63–1.80)	.828	1.33 (0.58–3.08)	.499	1.26 (0.63–2.52)	.516
High-risk antibiotics	1.06 (0.51–2.20)	.874	0.99 (0.48–2.04)	.980	0.93 (0.40–2.17)	.874
**Outcomes**						
Rehospitalization	**1.86 (1.05**–**3.27)**	**.032**	**3.00 (1.73**–**5.21)**	**<0.001**	1.62 (0.79–3.31)	.191
Clinical infection (any infection after first colonization)	1.40 (0.82–2.39)	.214	**2.37 (1.23**–**4.58)**	**0.010**	**1.69 (1.03**–**2.79)**	**.038**

Note. RRR, relative risk ratio; CI, confidence interval; PSMS, physical self-maintenance scales; LOS, length of stay; NH, nursing home. Bold indicates statistical significance.

Adjusted multinomial logistic regression analysis was conducted comparing transient R-GNB colonization to uncolonized status, persistent R-GNB colonization to uncolonized status, and persistent colonization versus transient R-GNB colonization (Table 3). Persistent R-GNB colonization was associated with lower age (OR, 0.98; 95% CI, 0.96–1.00; *P* = .020), higher PSMS (OR, 1.14; 95% CI, 1.05–1.23; *P* = .002), and longer lengths of nursing home stay (OR, 1.02; 95% CI, 1.01–1.02; *P* <.001) compared with uncolonized patients, adjusting for covariates. Transient R-GNB colonization was also associated with higher PSMS (OR, 1.10; 95% CI, 1.01–1.19; *P* = .023) compared with uncolonized patients.

**Table 3.** Adjusted Multinomial logistic regression for Transient and Persistent R-GNB Colonization

Variable	Transient vs Uncolonized RRR (95% CI)	*P* Value	Persistent vs Uncolonized RRR (95% CI)	*P* Value	Persist vs Transient RRR (95% CI)	*P* Value
Age	0.99 (0.97–1.00)	.128	**0.98 (0.96**–**1.00)**	**.020**	0.99 (0.97–1.01)	.395
Sex, male	0.73 (0.48–1.12)	.146	0.93 (0.51–1.69)	.819	1.28 (0.74–2.22)	.379
Race, nonwhite	1.62 (0.77–3.40)	.200	1.49 (0.58–3.84)	.407	0.92 (0.61–1.40)	.695
Charlson comorbidity index	1.02 (0.94–1.11)	.585	0.99 (0.92–1.08)	.885	0.97 (0.88–1.07)	.565
**PSMS score**	**1.10 (1.01**–**1.19)**	**.023**	**1.14 (1.05**–**1.23)**	**.002**	1.04 (0.94–1.14)	.478
Open wounds	1.61 (0.62–4.15)	.327	2.00 (0.73–5.49)	.179	1.24 (0.50–3.08)	.635
Duration of study follow-up, mean (SD)	**1.01 (1.00**–**1.02)**	**.030**	**1.02 (1.01**–**1.02)**	**<.001**	**1.01 (1.00**–**1.01)**	**.007**
High-risk antibiotics	1.00 (0.47–2.13)	.997	0.91 (0.46–1.79)	.779	0.91 (0.34–2.41)	.848

Note. R-GNB, resistant gram-negative bacteria; RRR, relative risk ratio; CI, confidence interval; PSMS, physical self-maintenance scales; SD, standard deviation. Bold indicates statistical significance.

## Discussion

In this retrospective study of ARO colonization status among a large longitudinal cohort of recently hospitalized nursing home patients, persistent R-GNB colonization was associated with lower age, lower functional status, and prolonged lengths of stay. Additionally, higher hospital readmission rates were seen in patients persistently colonized with R-GNB in unadjusted analyses. We did not detect an association between antibiotics or indwelling device use and persistence of R-GNB colonization. Resistance profiles varied from species to species; most *E. coli* and *P. mirabilis* were fluoroquinolone resistant, whereas *K. pneumoniae* exhibited relatively equal proportions of resistance to ciprofloxacin, ceftazidime, and imipenem.

Healthcare-acquired infections (HAIs) are associated with adverse clinical outcomes among nursing home patients; they are a major cause of hospital readmission among older patients in nursing home settings.^8^ ARO colonization rates in nursing homes are extremely high, and HAIs due to AROs, including R-GNB, are associated with particularly severe and poor outcomes.^9–12^ In this study, 47% of nursing home patients followed for ≥3 visits were persistently colonized with the same R-GNB, predominantly detected in rectal and groin body sites. These patients had significantly longer stays, with a median of 61 days, compared to 28.5 days for the entire nursing home population. *E. coli*, *Morganella morganii*, and *P. mirabilis* accounted for the longest periods of colonization. Prolonged R-GNB colonization is common among nursing home patients with dementia, with a median colonization duration of 211 days among a cohort of 33 nursing home patients as determined by collection of serial rectal swabs.^5^ Persistence of colonization is problematic for several reasons: (1) increased risk of HAI due to underlying R-GNB colonization^13,14^ and (2) potential for patient-to-patient R-GNB spread in settings with historically high rates of intrafacility transmission.^15^ Prior work has suggested that R-GNB are transmitted from patients to healthcare workers in ∼9% of interactions.^16^ Understanding factors associated with prolonged colonization will allow for addressing modifiable risk factors to reduce risk to the individual patient and to the nursing home population.

In this study, low functional status, as measured by PSMS, was associated with persistent R-GNB colonization. Prior work has shown high rates of R-GNB colonization among nursing home patients.^17^ Functional disability has been independently linked to shortened time to new ARO acquisition, including R-GNBs.^18^ This may be due to more frequent contact by healthcare providers or increased gut dysbiosis, leading to increased susceptibility to ARO acquisition. Little is known about variables that influence the duration of colonization; determining modifiable factors may allow for targeted interventions that attempt to promote decolonization of R-GNB. Such factors include utilizing enhanced barrier precautions for nursing care in patients with low function and augmented physical or occupational therapy services for debilitated patients with known R-GNB colonization.

Surprisingly, antibiotic use, including use of high-risk agents, a known risk factor for MDRO colonization, was not associated with persistent R-GNB colonization in this cohort.^19^ All 3 groups had 50%–60% antibiotic exposure over the prior 30 days, reflecting heavy antibiotic use in nursing-home populations with recent hospital admissions. Prior studies have found associations between MRDO colonization and chronic wounds, but this variable was not independently associated with persistence of R-GNB colonization, possibly due to inadequate power or interaction with low functional status. Additionally, on multivariable analysis, older age exhibited a slight protective effect against persistent R-GNB colonization. The significance of this finding is unclear. We suspect that multimorbidity and low function are much more important factors influencing the duration of colonization with R-GNB for this post-acute care population than age alone.

Persistent R-GNB colonization has potentially serious consequences at the individual patient level, with higher rates of hospital readmission compared to uncolonized and transiently colonized nursing home patients. It is unclear whether this association is related to higher rates of debility, longer lengths of stay, more frequent infectious complications, or other unknown factors.

This study had several limitations. By restricting the analysis to patients with ≥3 visits, the reduced size of our study population may have limited the ability to discern differences between the groups. Nevertheless, the large size of this cohort adds valuable information regarding factors related to persistence of R-GNB colonization among nursing home patients. Due to the imperfect sensitivity of body-site cultures, false-negative results may have occurred, leading to misclassification bias between the transiently and persistently colonized groups. However, we required negative cultures from multiple consecutive visits to classify a patient as transiently colonized, limiting this risk. We were unable to perform molecular analysis of R-GNB isolates over time to confirm that growth on consecutive cultures was due to persistent colonization rather than repeated acquisition of different strains of the same bacterial species. Lastly, we did not determine causality regarding prolonged R-GNB colonization in this study; association between specific factors and R-GNB carriage will require further study to determine whether modification of these factors influence the duration of colonization.

In conclusion, nursing home patients persistently colonized with R-GNB were more likely to have lower functional status, longer lengths of stay, and more frequent hospital readmissions. Antibiotic use and indwelling device use were not associated with R-GNB persistence. Further studies are needed to determine whether improving functional capacity could potentially reduce duration of R-GNB colonization.

## Supplementary material

For supplementary material accompanying this paper visit https://doi.org/10.1017/ice.2022.225. click here to view supplementary material
