# Supplementary material for: Epidemiology of resistant gram-negative bacteria in nursing homes
Source: Infect Control Hosp Epidemiol. 2023 Mar 14;44(9):1423–8. doi: 10.1017/ice.2022.225 (PMC10507500; doi:10.1017/ice.2022.225)
Supplement: Supplementary file 1 [file S0899823X22002252sup001.docx]

**Supplemental Table 1: Microbiology & Anatomical Sites of R-GNB Colonization**

|  | Total Colonized (n=169) | Transient R-GNB Colonization (n=89) | Persistent R-GNB Colonization (n=80) |
| --- | --- | --- | --- |
| **Patient Colonization** |  |  |  |
| *Escherichia coli* | 57 (33.7%) | 32 (36.0%) | 25 (31.3%) |
| *Proteus mirabilis* | 19 (11.2%) | 2 (2.3%) | 17 (21.3%) |
| *Klebsiella pneumoniae* | 18 (10.7%) | 5 (5.6%) | 13 (16.3%) |
| *Morganella morganii* | 13 (7.7%) | 6 (6.7%) | 7 (8.8%) |
| *Enterobacter cloacae* | 12 (7.1%) | 11 (12.4%) | 1 (1.3%) |
| *Acinetobacter baumanii* | 9 (5.3%) | 5 (5.6%) | 4 (5.0%) |
| *Enterobacter aerogenes* | 9 (5.3%) | 6 (6.7%) | 3 (3.8%) |
| *Pseudomonas aeruginosa* | 6 (3.6%) | 3 (3.4%) | 3 (3.8%) |
| *Stenotrophomonas maltophilia* | 4 (2.4%) | 3 (3.4%) | 1 (1.3%) |
| *Citrobacter koseri* | 4 (2.4%) | 3 (3.4%) | 1 (1.3%) |
| *Citrobacter freundii* | 3 (1.8%) | 1 (1.1%) | 2 (2.5%) |
| *Providencia stuartii* | 2 (1.2%) | 0 (-) | 2 (2.5%) |
| *Pantoea spp.* | 2 (1.2%) | 2 (2.3%) | 0 (-) |
| *Burkholderia cepacia* | 1 (0.6%) | 1 (1.1%) | 0 (-) |
| *Hafnia alvei* | 1 (0.6%) | 0 (-) | 1 (1.3%) |
| *Klebsiella oxytoca* | 1 (0.6%) | 1 (1.1%) | 0 (-) |
| *Photobacterium damselae* | 1 (0.6%) | 1 (1.1%) | 0 (-) |
| *Proteus vulgaris* | 1 (0.6%) | 1 (1.1%) | 0 (-) |
| *Pseudomonas spp.* | 1 (0.6%) | 1 (1.1%) | 0 (-) |
| *Pseudomonas luteola* | 1 (0.6%) | 1 (1.1%) | 0 (-) |
| *Unknown* | 4 (2.4%) | 4 (4.5%) | 0 (-) |
| **Body Sites colonized (initial colonized visit)** |  |  |  |
| Rectal | 86/130 (66.2%) | 39/68 (57.4%) | 47/62 (75.8%) |
| Groin | 73/169 (43.2%) | 39/89 (43.8%) | 34/80 (42.5%) |
| Oral | 32/161 (19.9%) | 14/85 (16.5%) | 18/76 (23.7%) |
| Hands | 23/168 (13.7%) | 9/89 (10.1%) | 14/79 (17.7%) |
| ≥ 2 body sites colonized | 50/169 (29.6%) | 19/89 (21.4%) | 31/80 (38.8%) |

**Supplemental Table 2: Resistance Profiles of Persistently Colonizing Gram-Negative Bacteria on First Detection**

|  | Ciprofloxacin-  Resistant | Ceftazidime-Resistant | Carbapenem-  Resistant |
| --- | --- | --- | --- |
| **Patient Colonization** |  |  |  |
| *Escherichia coli (N=25)* | 22 (88.0%) | 10 (40.0%) | 2 (8.0%) |
| *Proteus mirabilis (N=17)* | 17 (100.0%) | 1 (5.9%) | 0 (--) |
| *Klebsiella pneumoniae (N=13)* | 8 (61.5%) | 9 (69.2%) | 4 (30.8%) |
| *Morganella morganii (N=7)* | 3 (42.9%) | 4 (57.1%) | 7 (100.0%) |
| *Enterobacter cloacae (N=1)* | 0 | 1 (100.%) | 0 |
| *Acinetobacter baumanii (N=4)* | 4 (100.0%) | 4 (100.0%) | 3 (75.0%) |
| *Enterobacter aerogenes (N=3)* | 0 | 0 | 3 (100.0%) |
| *Pseudomonas aeruginosa (N=3)* | 1 (33.3%) | 0 | 3 (100.0%) |
| *Stenotrophomonas maltophilia (N=1)* | 0 | 1 (100.0%) | 1 (100.0%) |

**Supplemental Table 3: Duration of Colonization among Persistently Colonized Patients**

|  | N Patients persistently colonized with organism | Mean (range) duration | Median (IQR) duration |
| --- | --- | --- | --- |
| **Organism** |  |  |  |
| *Proteus mirabilis* | 27 | 73.9 (6-188) | 56 (15-121) |
| *Escherichia coli* | 32 | 61.6 (12-175) | 54.5 (28.5-85) |
| *Klebsiella pneumoniae* | 16 | 64.7 (7-187) | 37.5 (13.5-120.5) |
| *Morganella morganii* | 12 | 67.7 (12-167) | 46.5 (14.5-118.5) |
| *Pseudomonas aeruginosa* | 6 | 36.5 (14-64) | 31.5 (15-63) |
| *Acinetobacter baumanii* | 5 | 44 (6-95) | 47 (18-54) |
| *Enterobacter aerogenes* | 4 | 25.5 (14-41) | 23.5 (17.5-33.5) |
| *Enterobacter cloacae* | 3 | 62 (13-159) | 14 (13-159) |
| *Citrobacter freundii* | 3 | 41 (21-56) | 46 (21-56) |
| *Providencia stuartii* | 3 | 125.7 (42-188) | 147 (42-188) |
| *Stenotrophomonas maltophilia* | 2 | 7 (-) | 7 (-) |
| *Citrobacter koseri* | 1 | 14 (-) | 14 (-) |
| *Hafnia alvei* | 1 | 26 (-) | 26 (-) |
| *Enterobacter asburiae* | 1 | 25 (-) | 25 (-) |
| **Patient** |  |  |  |
| *Longest duration colonized with specific organism* | 80 | 61.6 (6-188) | 44.5 (15-90.5) |
